# Predicting Ligand Binding Modes by Scaffold-Guided Structure Refinement

**DOI:** 10.1021/acs.jmedchem.6c00089

**Published:** 2026-07-24

**Authors:** Jonathan Pletzer-Zelgert, Matthias Rarey, Bernd Kuhn

**Affiliations:** † 14915 University of Hamburg, ZBH - Center for Bioinformatics, Albert-Einstein-Ring 8-10, 22761 Hamburg, Germany; ‡ 1529 Roche Innovation Center Basel, F. Hoffmann-La Roche Ltd., Grenzacherstrasse 124, 4070 Basel, Switzerland

## Abstract

Efficient structure-based drug design relies on knowledge of a ligand’s binding pose and its specific interactions—information that is often not available experimentally. Despite the plethora of binding mode prediction methods—including cofolding—achieved accuracies are often insufficient. Here, we present “Scaffold-Guided Structure Refinement”, leveraging information on known binders within ligand series targeting a specific protein. Our method is based on the observation that shared molecular scaffolds among binders exhibit conserved binding modes. By applying molecular docking to diverse target model conformations, we identify those simultaneously allowing consistent scaffold placement, favorable interactions and low ligand strain. We demonstrate this approach’s ability to optimize models from different initial sources—including an inaccurate cofolding model—in three case studies. In all cases, we successfully identified critical induced fit effects and accurately reconstructed near-native ligand binding modes with scaffold root-mean-square deviation (RMSD) values of at most 2.2 Å.

## Graphical abstract

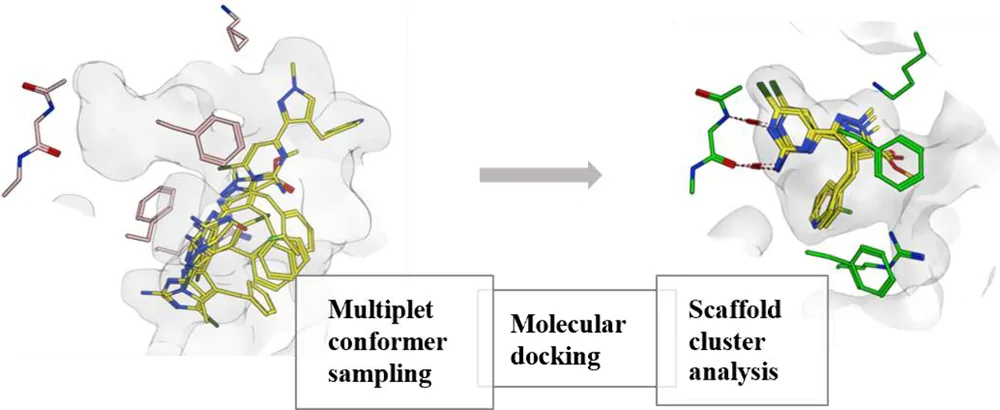

## Introduction

Structure-based design is a well-established approach for accelerating small molecule drug discovery.^1^ Ideally, several high-resolution X-ray crystal or cryo-EM structures of a ligand series bound to the target protein are available, enabling experts to optimize binding affinity while ensuring favorable molecular properties for a drug-like profile. However, for many pharmaceutical targets, this ideal scenario is unattainable, necessitating the use of computational models for protein-structure-guided ligand design. Even when experimental protein structures are available, predicting the binding mode of a ligand series can be challenging because of induced fit effects, which may significantly alter the shape of the binding site or the orientation of protein residues interacting with the ligand. Furthermore, many targets lack structural data entirely, requiring the construction of full protein or protein–ligand models. This process becomes particularly uncertain when no structural information from related proteins is available.^2^

Various computational techniques have been developed to aid in the construction of a protein–ligand binding model. Among the most fundamental is molecular docking, which involves fitting compounds into a protein structure or model. While docking can incorporate limited conformational sampling of preselected protein residues to account for potential induced fit effects, its success in capturing these effects is often limited and strongly dependent on the extent of flexibility considered.^3^ To improve upon this, resource intensive approaches such as induced fit docking combined with molecular dynamics simulations (IFD-MD) have been proposed.^4^ In cases where no experimental protein structure exists, homology modeling using templates from related proteins is a common strategy.^5^ Recent advances in deep learning-based protein structure prediction, such as AlphaFold,^6^ have significantly expanded the availability of structural models.^7^ These methods have now been extended to include cofolding of protein–ligand complexes.^8–11^ While promising, recent evaluations of cofolding methods suggest that their prediction accuracy is highly dependent on the similarity of input data to the training set, making extrapolation to novel protein–ligand interfaces unlikely to succeed.^12,13^ Additionally, Masters et al. investigated the effect of binding site mutations on poses predicted by AlphaFold3, revealing a lack of generalizability and insufficient consideration of protein–ligand interactions.^14^

Regardless of a model’s origin, its value for structure-based drug design during an active project will depend on the quality of molecular designs originating from it. Various studies, which aim to evaluate protein model quality, include molecular docking of known binders into models of the target and a manual analysis of the result’s accordance with experimental ligand data.^15–18^ When a target model is close to the native holo conformation, molecular docking is usually successful in determining a ligand’s binding pose.^19,20^ Therefore, successful docking of known binders can bear information on the protein model itself, and some approaches have previously aimed at exploiting this systematically in order to improve modeling of a target’s binding site. Evers et al. have shown that structural modeling of protein targets can be improved by the incorporation of ligands. Their MOBILE method refines initial homology models by docking active ligands and deriving distance restrains for further optimization.^21–24^ More recently, Karelina and Dror demonstrated that the systematic evaluation of docking poses can improve model selection from a variety of deep learning-derived structures.^25^ We believe that there is a need for a more generalizable method of this kind, assisting drug designers in addressing the model selection and induced-fit problem. All mentioned approaches either involve a significant amount of manual inspection or are not agnostic toward the method used to create the initial model. This new method should be applicable to a wide range of targets while reducing the need for computationally demanding methods like molecular dynamics simulations.

In this study, we propose a workflow, consisting of novel and previously established tools, for generating variations of initial protein models from external sources and ranking their quality by a ligand-informed approach. We call this methodology “Scaffold-Guided Structure Refinement” (SGSR), since it is based on two main assumptions. First, we assume that a series of known binders that share a common molecular substructure will adopt poses with a highly conserved binding mode of the shared scaffold. While this assumption will not always hold and there have been reported examples to the contrary for fragments,^26–28^ it is still commonly observed in structure-enabled discovery projects and one of the core principles behind structure-based lead optimization.^29–32^ Unfortunately, no systematic study has quantified the magnitude of scaffold displacements across protein–ligand systems. Indirect support for our hypothesis comes from studies on nonadditivity effects in structure–activity relationships (SARs).^33^ These were found to be rare and often explainable by local substituent effects. Since a non-conserved scaffold binding mode should manifest itself in nonadditive SAR, the rarity of such effects is consistent with the premise that significant scaffold displacements are uncommon. This is evident not only in more obvious cases, such as orthosteric protein kinase inhibitors, where the hinge binding motif is tightly anchored to the protein via multiple hydrogen bonds, but can also be observed in protein–ligand complexes with less directed scaffold interactions. One illustrative example is inhibitor series binding to the ligand binding domain of the Farnesoid X Receptor (FXR), a member of the nuclear hormone receptor family. As can be seen from the overlay of X-ray crystal structures in Figure 1A, three distinct inhibitor series—while binding to the same binding site region—have markedly different scaffold positions and lack any apparent shared pharmacophores. This variability is a result of the hydrophobic and flexible FXR binding site. In contrast, within each individual series, the experimental binding modes of the molecules show excellent alignment of their respective scaffolds (Figure 1B).

**Figure 1:**
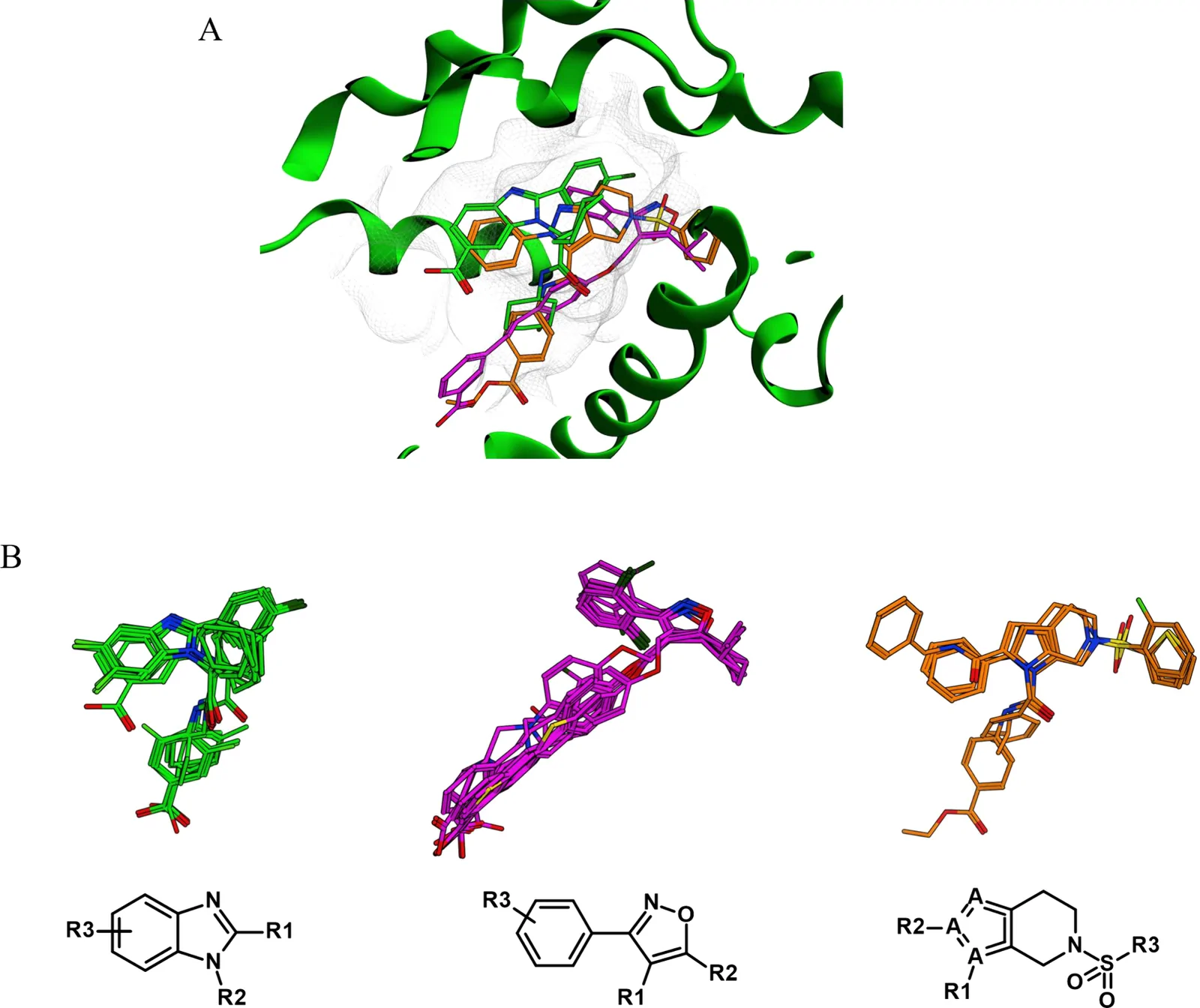
A) Overlay of the X-ray crystal structure determined binding modes of three FXR inhibitor series (benzimidazole, green, pdb code: 3OKH;^34^ isoxazole, magenta, pdb code: 3DCT;^35^ sulfonamide, orange, pdb code: 5Q0Q.^36^) B) Series-specific crystal structure overlays of binding modes in FXR (left: benzimidazoles, pdb codes: 3OKH, 3OKI, 3OLF, 3OMK, 3OMM, 3OOF, 3OOK;^34^ middle: isoxazoles, pdb codes: 3DCT, 3DCU,^35^ 3HC5, 3HC6,^37^ 3RUT, 3RUU,^38^ 7VUE;^39^ right: sulfonamides, pdb codes: 5Q0Q, 5Q0R, 5Q0T^36^).

The second assumption is that a state-of-the-art docking algorithm will be able to create a correct protein–ligand pose, if the protein model is sufficiently close to the native holo conformation of the complex.^40,41^ From these two principles, we derive the hypothesis that performing docking with a series of binders to a near-native protein model will result in highly similar scaffold poses, while models which deviate further from it will lead to a more distributed placement. The SGSR is aiming to exploit this by performing geometric density clustering on its docked ligand poses and using this information to prioritize protein–ligand models.

An obvious prerequisite for this method is the presence of a sufficiently large congeneric ligand series. While this is often given in industrial drug design projects—and therefore a realistic scenario—the amount of publicly available data sets fulfilling this criterion is limited.^29^ Furthermore, testing SGSR in a way that mimics active drug design projects requires the initial structure models to reflect the quality such a model would realistically have. This limits the number of cofolding models suitable for analysis, since we have to exclude all target-ligand combinations that were included in the training sets of these models.^12–14^ Therefore, this study will be limited to three application scenarios covering different target classes and model origins. We apply SGSR to binding mode prediction challenges that are all relevant for structure-based drug design. First, we simulate a project scenario where an X-ray crystal structure of the target protein Fatty Acid Binding Protein 4 (FABP4), is available, but the binding mode of a ligand series cannot be reliably modeled. Second, we evaluate the capability of our approach to predict the binding mode of a ligand series within sets of homology models of phosphodiesterase 10A (PDE10). Third, we explore its utility as a postprocessing approach to refine binding mode predictions for adenylate cyclase 10 (ADCY10) generated by the BOLTZ-1 cofolding algorithm.^12^

## Results and Discussion

Our “Scaffold-Guided Structure Refinement” workflow starts from one or more initial protein models and involves the following key steps: (a) automatic or user-defined pocket detection, (b) rotamer multiplet detection combined with extensive conformational sampling to generate a diverse set of candidate binding sites, (c) docking of a ligand series into each of the binding sites, and (d) scaffold placement analysis and model scoring. This process is separated into two parts as illustrated in Figure 2. Part A includes steps for the generation of rotamer variants of initial models, while part B includes all steps for ligand docking, pose clustering and subsequent ranking of models.

**Figure 2:**
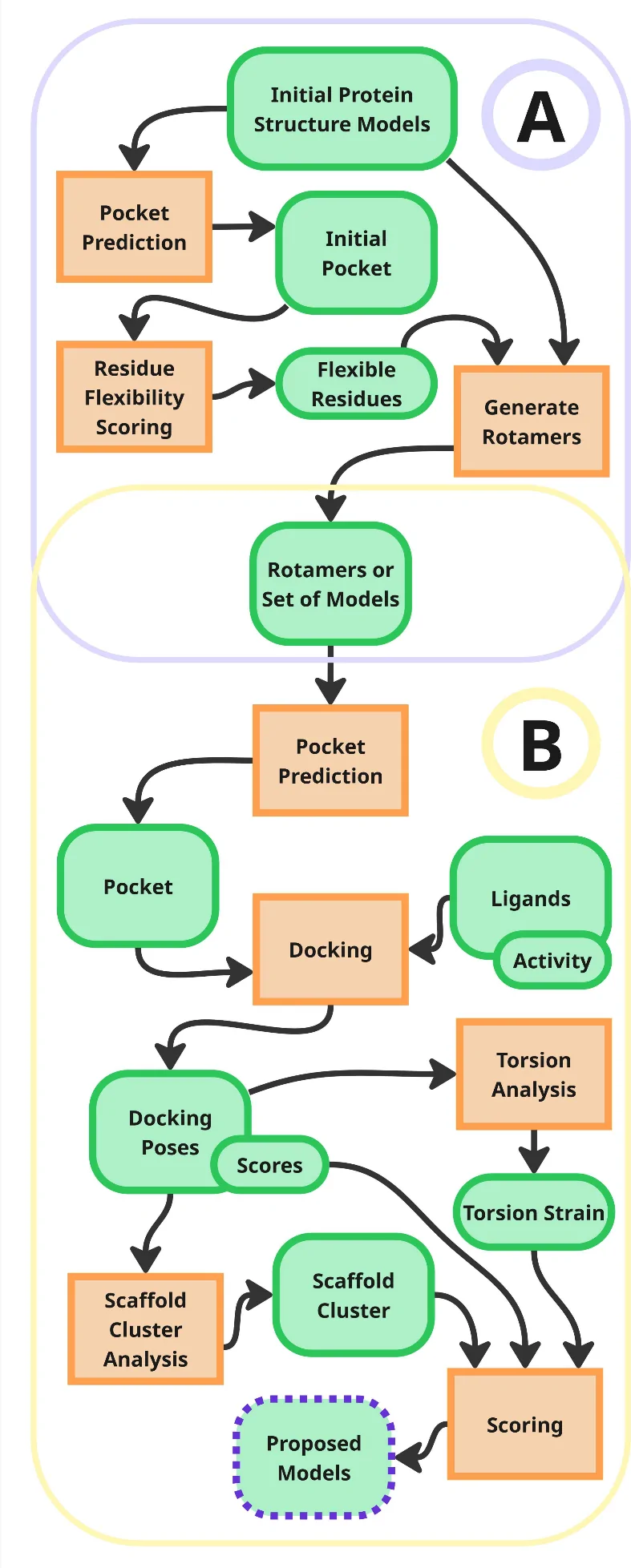
A schematic depiction of the two SGSR workflow parts. A) A set of initial protein structure models is subjected to a pocket prediction routine if the binding site is unknown. Subsequently, our novel tool for side chain flexibility scoring is used to generate sets of residues that are then fed into the rotamer generation tool. The result is a set of rotamer variants for each initial model. B) As in part A, a pocket is calculated for each model. These are then used for docking a set of known small molecule binders to the target. Resulting poses with their corresponding scores are analyzed in a novel scaffold clustering tool and checked for ligand strain.^42–44^ From these findings, a ranking of promising models is generated, which suggests models for further consideration.

Table 1 lists all specific tools used in our workflow and specifies whether we used pre-existing software or developed them for this work. All modules are part of the NAOMI ChemBio Suite and are free for academic use. They all run on standard CPU, and their runtimes are in the order of seconds, with the exception of JAMDA docking, for which 1–3 CPU minutes can be expected per single ligand docking.^45^

**Table 1:** Tools Used in This Work

Process	Used Tool	Developed for this work	Remark
Pocket Prediction	DoGSite3 3.0 ^46^	No	Used in both workflow parts A and B
Residue Flexibility Scoring	SideChainSelector 1.0	Yes	Based on preexisting Dunbrack Rotamer library^47^
Rotamer Generation	SideChainRotator 1.0	Yes	Based on preexisting Dunbrack Rotamer library^47^
Docking	JAMDA 1.2 ^41,45^	No	Softened clash potential was used
Torsion Analysis	TorsionAnalyzer 2.1 ^42–44^	No	
Scaffold Cluster Analysis	ScaffoldPlacement 1.0	Yes	
Comparison with Experimental Structures	BindingSiteComparer 1.0	Yes	

In the following, we highlight specific parts of the SGSR workflow, while a more detailed description of every step—especially for tools originating from this work—can be found in the Experimental Section. In general, part B can be used without the generation of rotamers, as long as a sufficiently large set of initial protein models is supplied. The method is agnostic to the origin of the models and could, in principle, be used to prioritize a large number of initial predictions from sources such as deep learning methods or frames of an MD simulation. Since SGSR is designed to improve models from various origins, direct comparison to established methods is somewhat challenging. Our application scenarios either employ cofolding or rely on legacy projects predating these methods; such comparisons would be problematic, since cofolding training data now encompasses these structural series.^12,48,49^ We therefore benchmark SGSR against side-chain flexible docking, a current standard method for addressing the conformational sampling challenge that SGSR aims to improve. All examples discussed in this work assume the standard scenario: the presence of one or more initial protein models and known binders sharing a common molecular scaffold, but no information on the binding mode of the series or the binding pocket.

### Creating Rotamer Variants of Starting Models

Starting protein models are subjected to pocket detection using the geometric pocket detection tool Dogsite3.^46^ The resulting top-ranked pockets are scored by our novel flexibility selection tool described in the Experimental Section. It provides suggestions for defining flexible side-chain sets based on solvent exposure and structural diversity of constructible rotamer conformations. A previous study by Najmanovich et al.^50^ demonstrated that 85% of induced fit effects can be accurately captured with three or fewer side-chain rotations, which is why, by default, we select at most four in a single set. These sets are then used for rotamer construction using a novel tool based on the backbone-dependent Dunbrack rotamer library.^47^ The resulting models serve as the input for part B.

### Creating Clusters of Scaffold Poses

The pool of models—which is significantly enlarged by the generation of rotamer variations—is subjected to a new iteration of Dogsite3 pocket predictions, since the rotation of side chains can potentially influence pocket size. Ligands in the series are prepared using UNICON^51^ to ensure a consistent format and are subsequently docked into each of the generated pockets with the highly automated docking tool JAMDA.^41,45^ We also explored docking methods that allow for limited side chain flexibility during pose optimization, such as GOLD^52,53^ (see Comparison with Side Chain Flexible Docking). However, we found that such methods typically produced diverse protein–ligand conformations for different members of the ligand series, often accompanied by a higher variation in scaffold poses. As a result, we decided to keep receptors rigid during docking.

From all resulting docking poses, we select the top-scoring pose for each ligand-model pair, which is then used for subsequent scaffold analysis. This analysis is performed with the new tool for scaffold clustering described in the Experimental Section, which utilizes the clustering method DBSCAN^54^—with the root-mean-square deviation (RMSD) as distance—to identify scaffold poses that frequently occur in the docking results. The DBSCAN algorithm is a density-based clustering algorithm that separates poses into clusters if sufficiently many have low distances to each other, and classifies poses without such neighbors as noise. Thereby it directly generates candidate clusters which can be ranked in the next step. While choosing only a single pose per ligand will discard some high-ranked near native poses, we still found this approach sufficient for all investigated targets, while reducing the amount of non-native clusters identified. For cases with smaller ligand series—where it might not be acceptable to exclude any ligands during clustering—it might be helpful to use more docking poses for the subsequent clustering.

All tools—existing or newly developed—are based on the NAOMI library and its internal chemistry model.^55,56^

### Ranking Resulting Models

As we do not have a large data foundation in this work, we aimed to develop a simple ranking method in line with expert intuition. Our main objective for model ranking is that a good model can be identified within the top 5 solutions, which is a reasonable number for visual inspection by an expert.

When inspecting clusters of scaffold poses and their corresponding docking results, we found that models which lead to a near-native ligand pose indeed exhibit scaffold clusters containing most ligands of the series. However, we also observed some scaffold clusters covering a large number of ligands in non-native poses for models that differ from experimental structures. In these cases, we found that either relative docking scores were significantly worse than for near-native poses or that ligand poses were exhibiting substantial ligand strain. In such clusters, high-energy ligand conformations were artificially stabilized by high-scoring interactions of the scaffold. These observations led to the following ranking procedure.

First, we discard ligand clusters with strained torsion profiles as detected by the Torsion Analyzer tool.^42–44^ For each cluster, we determine its set of ligands with one or more torsion angles falling into the worst category “strained”. If 20% or more of the poses in a cluster exhibit strained torsion angles—a cutoff derived from in-house routine praxis among experts—the cluster is discarded. We have not observed desirable ligand pose clusters which were filtered out by this criterion. The importance of this step is illustrated for the PDE10 case in Figure 3.

**Figure 3:**
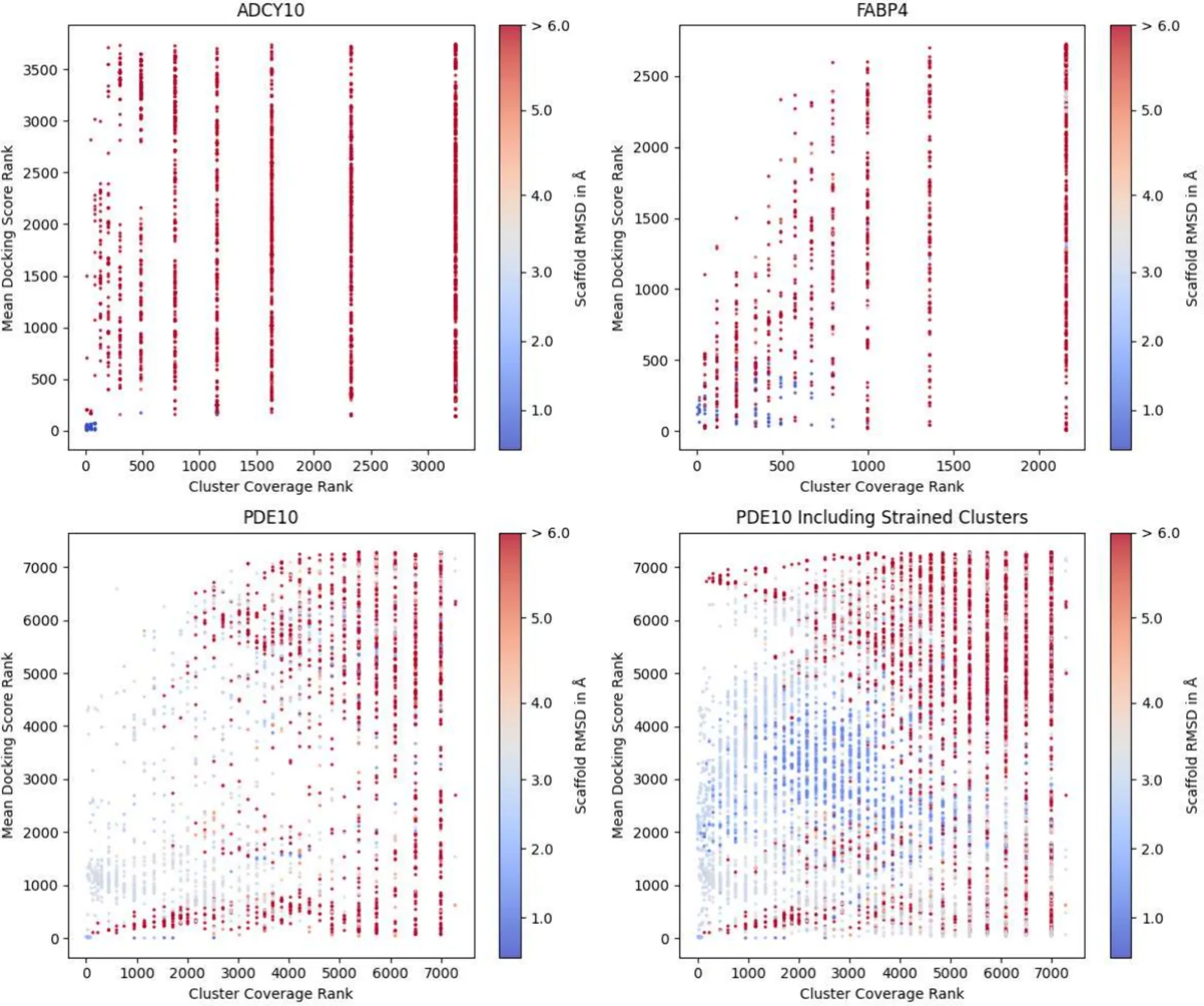
Scatter plots for all three different targets with both ranks that we use in combination for model ranking (cluster coverage and mean docking score). The RMSD of the clusters’ scaffold representative to the pose from an X-ray crystal structure is shown in a red/blue color gradient. RMSD values above 6 Å are colored uniformly red. ADCY10: We find an almost ideal ranking with almost all clusters of low RMSD showing high ranks regarding cluster coverage and mean docking score; FABP4: High ranking models are strongly enriched at the top, but we also find negative cases that are ranked highly; PDE10: Top ranked models exhibit scaffolds with RMSDs around 2.5 Å. Visual inspection reveals that the bicyclic aromatic scaffold is appropriately positioned but is regularly flipped by 180 deg without affecting key interactions. Considering the strong shape overlap and preserved interactions, we still regard these cases as successes and assess the ranking performance as satisfactory. On the lower right side, the distribution without filtering of clusters with strained poses is shown for PDE10. PDE10 exhibits an especially high number of scaffold poses with low RMSD that are only possible due to strained torsion angles. Most top ranked models are unaffected by this filtering step.

Subsequently, we use two descriptors to rank each cluster based on the previously described observations. One is the percentage of ligands being part of the cluster (cluster coverage), and the other is the mean docking score of its ligands. We consider the relative rank of these values compared to those of other clusters and sort the models with respect to the sum of these two ranks. Adding other descriptors, like the standard deviation of poses within a cluster, did not improve the ranking, nor did the usage of simple regression methods.

Scaffold RMSD values used for quality assessment were calculated with a newly developed utility tool (see Experimental Section), which compares each cluster’s representative scaffold pose with an experimental ligand pose after pocket alignment.

Figure 3 shows the distribution of these ranks in relation to the RMSD of the cluster’s scaffold representative to an experimental structure pose for our three application scenarios: FABP4, PDE10, and ADCY10. For PDE10 and ADCY10, we find that all top 5 models show a well positioned scaffold combined with crucial side chain rotations, which will be discussed in the next sections. For FABP4, we find that the model at rank 3 resembles the experimental structure while the other top 5 models are not considered a success. A complete precision-recall plot for each example can be found in the .

### Application Scenarios

#### FABP4

A common challenge in structure-based drug design remains the reliable modeling of binding modes of a ligand series of interest despite the presence of an experimental structure for the target protein. This could be because the ligand series preferentially binds to a protein conformation that is absent in the existing biostructures. In our first SGSR case study, we investigated such a scenario in a Roche legacy project focused on developing Fatty Acid Binding Protein 4 (FABP4) inhibitors against type II diabetes.^57^ Several X-ray crystal structures for this protein were available in the project, but the binding mode of a ligand series containing a triazolopyrimidinone core (Figure 4A) was unclear at that time.

**Figure 4:**
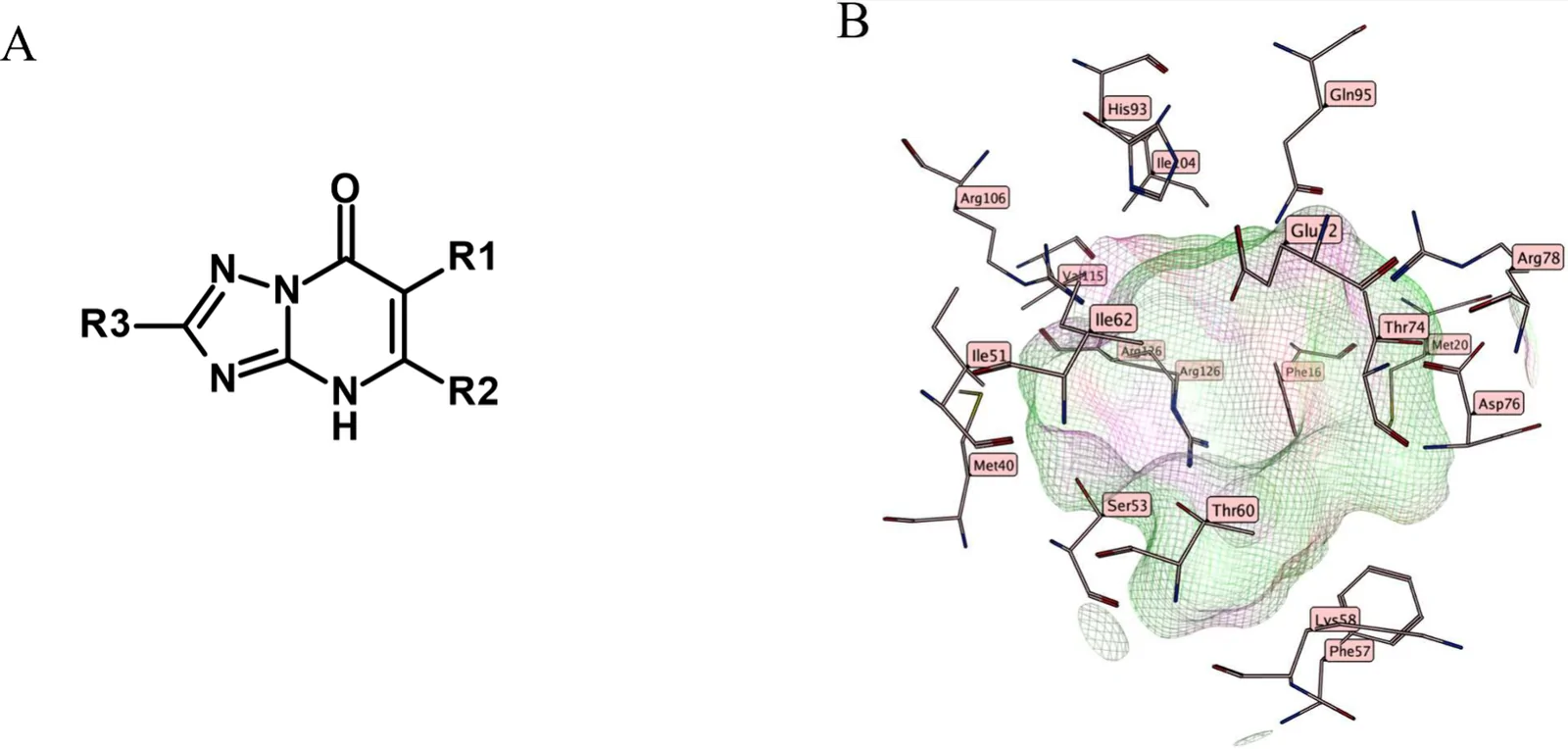
A) Triazolopyrimidinone series used in the FABP4 case study. The 17 ligands fabp4_1-fabp4_17 are listed in the . B) Illustration of the FABP4 binding pocket used as input (pdb code: 7FVU) and of the FABP4 residues sampled in the workflow. The multiplet combinations are summarized in Table 2.

Starting from one of the available FABP4 protein structures (pdb code: 7FVU), the side chain selection tool identified in total 19 amino acids around the ligand binding site (Figure 4B) and suggested the multiplet combinations summarized in Table 2 for rotamer sampling.

**Table 2:** List of Sampled FABP4 Side Chain Multiplets

FABP4 residue ids	# of conformers
[Met40,Ser53,Lys58,Thr60]	[7,3,9,3]
[Ser53,Phe57,Lys58,Thr60]	[3,4,9,3]
[Glu72,Thr74,Arg78,Ile104]	[7,2,9,5]
[Ile104,Arg106,Val115,Arg126]	[4,5,7,7]
[Ile51,Ile104,Arg106,Arg126]	[5,7,2,7]
[Thr74,Asp76,Arg78,Gln95]	[2,6,9,7]
[Met40,Ile51,Ile62,Arg106]	[7,4,4,7]
[Ile51,Ile62,Glu72]	[4,4,7]
[Ile62,Glu72,His93,Gln95]	[4,7,5,7]
[Phe16,Met20,Arg126]	[6,5,7]

Applying the full workflow on these 10 multiplet combinations yielded a total of 1,062 different FABP4 protein models that were ranked as described in the previous section. The summary table in the shows the ranked model list with annotations of different score contributions as well as the ligand and active site RMSD values compared to the FABP4 X-ray cocrystal structure with compound fabp4_16 (pdb code: 7FYJ), which was determined later in the project. The protein–ligand model ranked at position 3 showed a low scaffold RMSD of 0.84 Å as well as an active site RMSD of 0.56 Å, suggesting a good scaffold placement and protein–ligand model.

The placement of the triazolopyrimidinone scaffold in this binding model together with the rotated side chains [Met40, Ser53, Lys58, Thr60] compared to the input model is shown in Figure 5A. Of relevance is the concerted side chain movement of Met40 and Ser53, which opens a small pocket in FABP4 that can now accommodate the R3 substitutions on the scaffold. Fifteen out of 17 molecules could be docked into this protein model with the same scaffold placement yielding an excellent cluster coverage of 88% (Figure 5B). The mean JAMDA score in the cluster was −3.3 indicating good protein–ligand interactions in these models, and 12 out of 15 docked ligands (80%) had no strained torsions. An overlay of the suggested model for fabp4_16 with its X-ray cocrystal structure (Figure 5C) shows very good agreement in scaffold placement, ligand position and interactions, and particularly the presence of the induced fit pocket created by the rotation of the Met40 side chain. Such a model of correct scaffold placement, if available at the time, would have provided an excellent basis for structure-based design suggestions.

**Figure 5:**
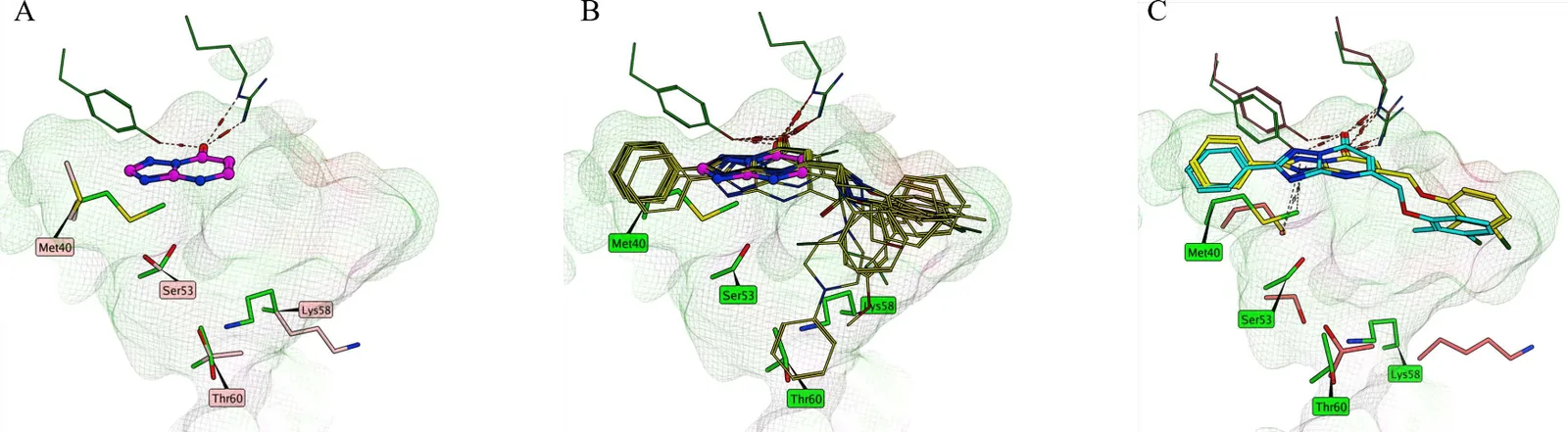
A) Triazolopyrimidinone scaffold (magenta) placement in one of the top-ranked FABP4 binding mode models (Rank 3, Model 16_Met40 _8_Ser53_0_Lys58_50_Thr60_1). Residues that were sampled in this model are highlighted in stick mode (input model: salmon, final model: green). The scaffold interacts through hydrogen bonds (red, dashed lines) with FABP4 residues Arg126 and Tyr128. B) Overlay of the docking poses of the 15 molecules (yellow) that are found in the top-ranking scaffold cluster. C) Overlay of the binding mode model of compound fabp4_16 in the proposed model (green, ligand: yellow) with its FABP4 cocrystal structure (red, ligand: cyan; pdb code: 7FYJ).

#### PDE10

A typically more difficult challenge exists when the ligand binding mode is unclear, and no prior structural information is available for the target of interest. In such cases, homology modeling using a structurally related protein template is a common approach to generate an initial target model. We simulated such a scenario for the SGSR approach by attempting to identify the experimental binding mode of a series of phosphodiesterase 10A (PDE10) inhibitors, starting from homology models based on the related PDE5 protein (sequence identity PDE10/PDE5: 29.1%). The inhibitor series featured a triazolopyrazine core (Figure 6A) with substitutions at four positions (R1–R4), and with the primary variation occurring at R4.^48^ It is important to note that the ligand series had pseudosymmetry in the triazolopyrazine core, where 57 of the 59 molecules had a consistent substitution pattern (R1 = R3 = methyl, R2 = hydrogen). To account for this, we applied a larger clustering threshold of RMSD = 2.0 Å instead of the standard value of 1.0 Å. We generated five homology models, each based on a distinct PDE5 template, and sampled five side chain multiplet combinations for each of the input models (). This yielded a total of 2,248 protein models, which were evaluated by our method.

**Figure 6:**
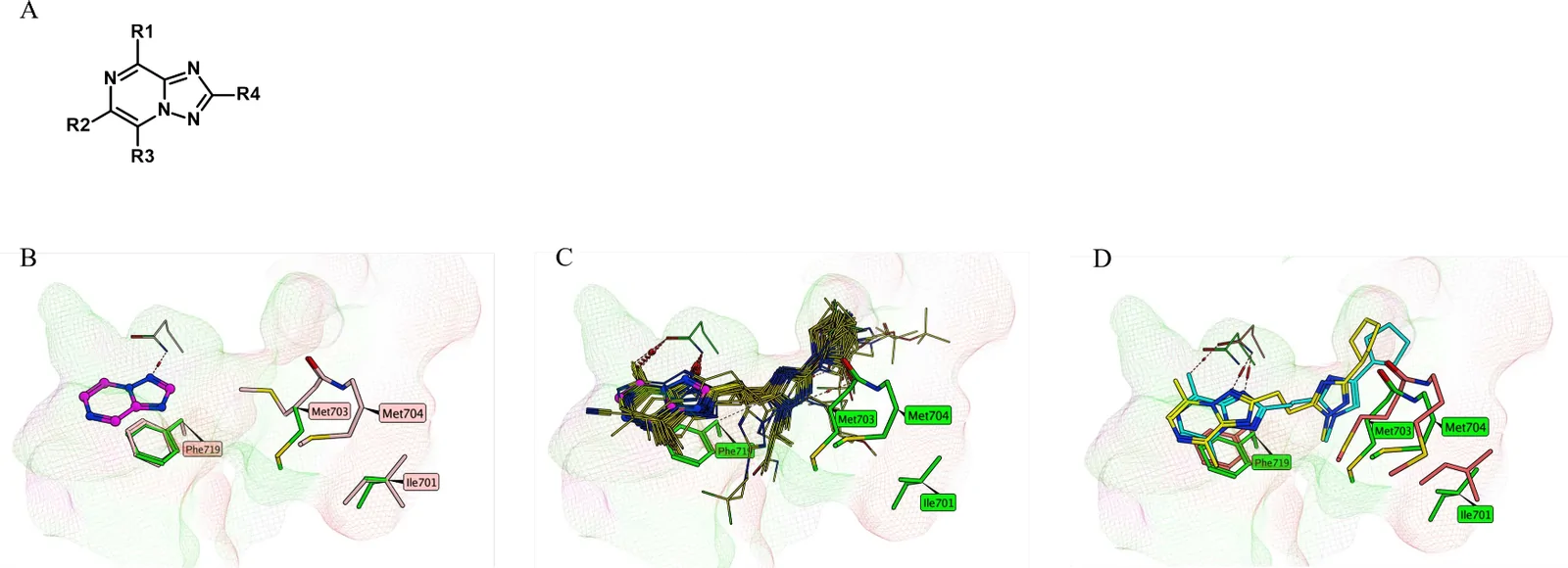
A) Triazolopyrazine series used in the PDE10 case study. The 59 ligands pde10_1-pde10_59 are listed in the . B) Triazolopyrazine scaffold (magenta) placement in the top-ranked PDE10 binding mode model (110_1t9s_Ile701_1_Met703_8_Met704_27_Phe719_0). Residues that were sampled in this model are highlighted in stick mode (input model: salmon, final model: green). The scaffold interacts through a hydrogen bond (red, dashed line) with PDE10 residue Gln716. C) Overlay of the docking poses of the 51 molecules (yellow) that are found in the top-ranking scaffold cluster. D) Overlay of the binding mode model of compound pde10_15 in the proposed model (green, ligand: yellow) with its PDE10 cocrystal structure (red, ligand: cyan; pdb code: 5SEP).

The top-scoring binding mode model showed a hydrogen bond of one of the aromatic nitrogen atoms of the core with the PDE-invariant side chain of Gln716 (Figure 6B). This model also revealed an outward movement of the Met703 side chain, creating an extended binding pocket along the R4 exit vector. This conformational change was critical for docking 86% of the ligand data set (51 out of 59 ligands) with a conserved scaffold placement in the model (Figure 6C). The 51 docked ligands in this cluster exhibited numerous favorable protein–ligand interactions (mean JAMDA score: −3.16) and 94% of them had no ligand strain (48 of 51 ligands) resulting in a Rank 1 assessment. An overlay of the predicted PDE10 binding mode of compound pde10_15 with its X-ray cocrystal structure (Figure 6D) showed excellent agreement in ligand placement, core interactions, and key side chain positions. The scaffold and active site RMSD values were 2.2 Å and 1.4 Å, respectively. The relatively high scaffold RMSD can be attributed to the above-mentioned pseudosymmetry of the triazolopyrazine core. Docked molecules in the top-ranked cluster exhibited both orientations of the core (Figure 6C), differing in the position of the nitrogen atom between R1 and R2. Importantly for the model and its use in design, both orientations satisfied key molecular recognition motifs around the core, which are hydrogen bonding with Gln716 and π–π stacking with Phe719.

#### ADCY10

The cofolding of protein–ligand complexes using deep learning methods has recently garnered significant attention.^8–11^ Although promising, different studies have demonstrated that the prediction accuracy declines for systems that differ from the training set and that specific protein–ligand interactions are frequently not well captured.^12,14^ In our study, we explored whether the SGSR workflow could serve as a postprocessing tool to enhance the accuracy of initial cofolding models. The system we examined was adenylate cyclase 10 (ADCY10), which exhibited poor performance in the Boltz-1 prediction for an aminopyrimidine ligand (Figure 7A). A set of 15 congeneric ligands with an aminopyrimidine scaffold (Figure 7B) was identified from ChEMBL.^58–61^

**Figure 7:**
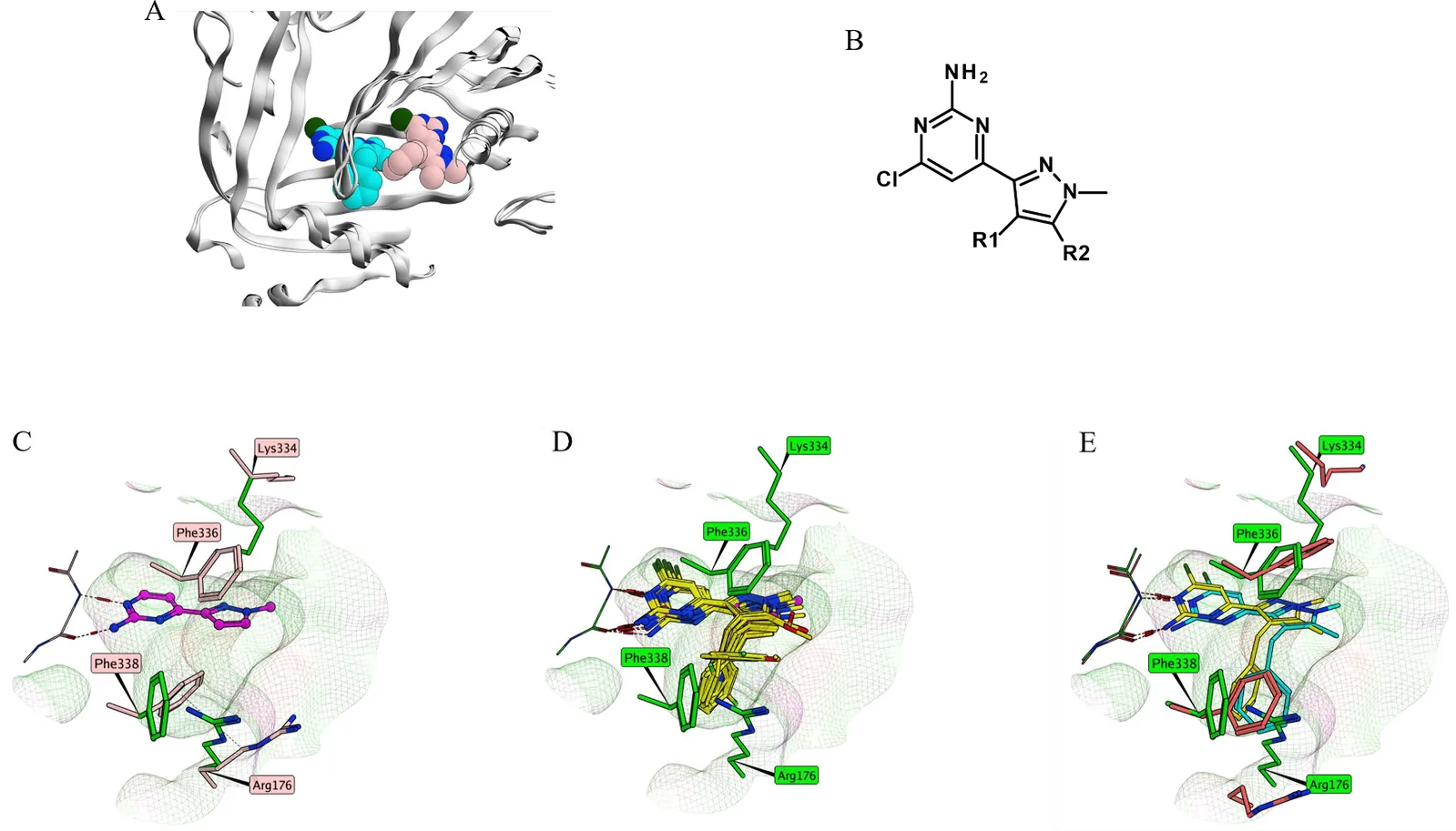
A) Overlay of the ligand binding mode in the protein ADCY10 from X-ray crystal structure (pdb code: 7OVD, cyan) with the Boltz-1 prediction (salmon).^12,62^ The ligand RMSD is 4.1 Å. B) Aminopyrimidine series used in the ADCY10 case study. The 15 ligands adcy10_1-adcy10_15 are listed in the . C) Aminopyrimidine scaffold (magenta) placement in the top-ranked ADCY10 binding mode model (114_Arg176_5_Lys334_16_Phe336_18_Phe338_6). Residues that were sampled in this model are highlighted in stick mode (input model: salmon, final model: green). The scaffold interacts through a hydrogen bond (red, dashed line) with the backbone of ADCY10 residue Val167. D) Overlay of the docking poses of the 14 molecules (yellow) that are found in the top-ranking scaffold cluster. E) Overlay of the binding mode model of compound adcy10_13 in the proposed model (green, ligand: yellow) with its ADCY10 cocrystal structure (red, ligand: cyan; pdb code: 7OVD).

We used the inaccurate Boltz-1 protein–ligand model as a starting structure for our workflow. After removing the ligand, 18 residues lining the ligand-binding pocket were automatically identified () and the 10 ADCY10 side chain multiplets listed in were conformationally sampled in 10 independent runs. This led to 2,152 protein models for scoring evaluation.

At Rank 1 of our model list, we identified a scaffold binding mode featuring two hydrogen bonds of the aminopyridine fragment to the Val167 backbone atoms of ADCY10 (Figure 7C). Fourteen out of the 15 ligands in the aminopyridine series could be successfully docked with conserved scaffold placement into this model (cluster coverage: 93%), with a favorable mean JAMDA score of −3.44 (Figure 7D). Furthermore, only one of the 14 ligands in this cluster exhibited ligand strain, further supporting the quality of our model. Comparison of the binding mode of the ligand from the cocrystal with the prediction revealed excellent overlap, with scaffold and active site RMSDs of 0.43 Å and 1.49 Å, respectively. The key conformational change enabling the docking of the congeneric series was an outward movement of Phe338, which, in the Boltz-1 prediction model, had restricted the binding pocket, making it too small for these compounds. The successful application of SGSR as a postprocessing method for refining initial cofolding methods is promising. A more comprehensive evaluation of this approach is underway and will be addressed in a future publication.

### Comparison with Side Chain Flexible Docking

To verify that our SGSR workflow has advantages over established methods, we performed side chain flexible docking with GOLD.^52,53^ The question at hand was whether this approach would lead to consistently placed ligands, even though the flexibility of specific side chains was considered independently for each ligand. For this, we defined a limited number of flexible side chains, which were subjected to optimization during molecular docking. We used the same initial starting models as for our SGSR approach with the exception of PDE10, for which we only used the best initial homology model which was derived from PDE5 template 1T9S. For each model, we performed one run with a broad selection of ten flexible side chains, as well as a narrower selection using only the side chains we knew would play a role in the induced-fit process. A list of them can be found in . We subsequently analyzed the output with the BindingSiteComparer tool for calculating the RMSD of the shared molecular scaffold of the series. As can be seen in Figure 8, the vast majority of all ligands could not be placed in a near native conformation, with only a single ligand being placed within 2 Å for the FABP4 focused run and one for each of the PDE10 runs. In general, the results showed no consistent protein–scaffold pose for these series and the outcome did not lead to a valid protein–ligand conformation. We thereby conclude, that incorporating side chain flexibility during docking alone will not enable the identification of valid protein ligand poses for our case studies.

**Figure 8:**
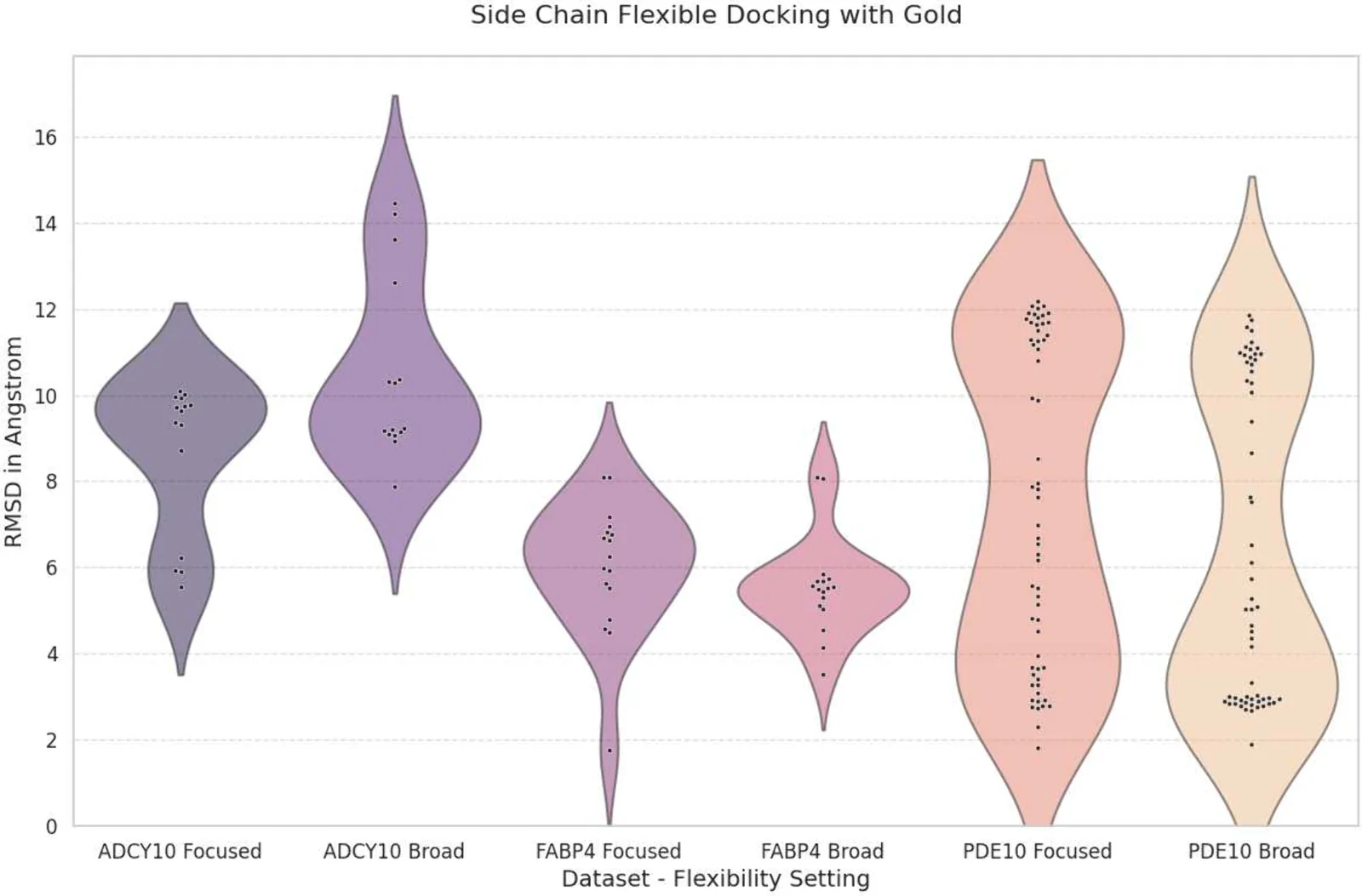
A violin plot containing all scaffold RMSD values for top scored docking poses from flexible GOLD docking. See for a detailed listing of flexible side chains during these experiments.

## Conclusions

In this work, we introduced “Scaffold-Guided Structure Refinement” as a novel approach for predicting binding poses of ligand series and their corresponding protein conformations. Our approach is based on extensive conformational sampling of automatically detected or user-defined side chain combinations to generate a large set of possible binding sites. It then leverages the common observation that ligands within a series typically share a conserved binding pose for their molecular scaffold. We employ the consistent placement of this scaffold during molecular docking, together with protein–ligand interaction scores and ligand strain analysis, as a quality measure for evaluating protein models. We demonstrated the capabilities of this method through three case studies, which encompassed different sources of starting models and different target classes. In these studies, we successfully identified induced fit effects and reproduced near-native ligand binding modes. The combination of geometric scaffold pose clustering and docking scores proved to be a reliable and interpretable measure for ranking the resulting models. While further application of the method is necessary to conclusively verify its generalizability, we found it to work consistently in all cases where model sampling included suitable target conformations, and are therefore optimistic that these results are transferable to other protein classes.

We recommend applying SGSR in projects where initial protein models have a reasonable fold but fail to explain the binding of known active ligands. Models of greater uncertainty might necessitate a broader sampling of backbone conformations, which is beyond the scope of this work. Many of the ligands in the discussed use cases exhibit strong binding to the target, suggesting optimized protein–ligand interactions. It remains to be seen whether the method performs equally well in scenarios where only weak binders are available. We are currently investigating the approach across different protein systems and ligand sets, spanning various affinity ranges.

Since the method requires various computational steps, one of our current objectives is to enhance automation to minimize the required manual effort. Furthermore, we aim to expand the amount of ligand information used for model evaluation. For instance, incorporating SAR data could provide a more detailed analysis of favorable interaction patterns within a ligand series. A reliable binding mode model should be able to explain most of the experimentally observed activity cliffs, and this could be incorporated into the ranking of the models. Additional strategies that could be used for the postprocessing and validation of our models include stability testing using MD simulations or free energy calculations to assess the correlation with experimental SAR.

## Experimental Section

### Pocket Detection

The first step in both SGSR workflow parts in Figure 2 is the calculation of druggable pockets, for which we use the tool Dogsite3.^46^ Dogsite3 is a purely geometric pocket detection approach based on a difference-of-Gaussians grid filter,^63^ which will score highly for enclosed regions. Among its different potential output formats, we process the identified pockets in the form of PDB files.^64^

For our application, we changed Dogsite3’s map cutoff parameter from −0.1 to −0.04, which results in bigger binding pockets. This adjustment accounts for the greater uncertainties inherent in our models, as Dogsite3 was originally parametrized using experimentally solved structures. However, due to the small number of examples available for this study, we did not perform a detailed optimization of this parameter, and for the application on new targets, we suggest that calculated pockets are visually inspected for the initial model(s).

Note that the size in the resulting pocket in part B may change due to the employed side chain rotation, and it is therefore recommended to subsequently recalculate the pocket. This is, for example, relevant in the PDE10 use case, where a methionine rotation opens up a side pocket that is crucial for binding of the ligand series.

### Rotamer Variants Generation of Initial Models

In general, we assume that SGSR can improve a structural model with a reasonable fold that lacks a few crucial side chain conformations. However, if all initial models strongly deviate in their backbone arrangement from the experimental structure, we do not expect to find a good result.

Therefore, our focus lies in identifying a set of side chains that have the potential to undergo relevant changes, and generating a sufficiently large set of rotamer variations from these. To this end, we developed two applications: one for scoring side-chain flexibility and another for generating rotamer variants of a given model. Both tools are based on the NAOMI library and its internal chemistry model,^55,56^ as well as the Dunbrack rotamer library.^47^ The Dunbrack rotamer library provides empirically observed, backbone-dependent side-chain conformations for all standard amino acids.

Since our primary interest lies in conformations relevant to ligand binding events, we consider the specific probabilities of rotamer occurrence in the library to be less representative for our application. Instead, we analyze the full set of stored conformations, exclude those that would result in obvious steric clashes, and cluster the remaining ones to select a diverse and manageable subset.

Generally, these applications aim to efficiently mimic expert intuition, and they have been developed in close consultation with such users. At the same time, they allow for additional manual specifications, such as adding or omitting residues and adjusting clash function parameters. For all examples investigated in this study, the default workflows, produced the presented results without further modifications.

#### Protein Side Chain Clashes

In order to exclude rotamer conformations that result in steric clashes, we incorporated a clash test after every conformational change. However, given that we are working with imperfect models, we decided to use the lenient clash term. We consider a certain amount of overall clashes acceptable as the model may still include a crucial side chain rotation. We also expect to perform a large number of these operations and therefore decided to use a simplified, linear clash term shown in eq 1.

To scale the combined van der Waals radius *r*_a,b_ of two atoms *a* and *b*, we define two parameters *c*_0_, *c*_1_ ∈ [0, 1] with *c*_1_ ≤ *c*_0_.^65^ If the distance between the atom centers *d*_*a*,*b*_ satisfies *d*_*a*,*b*_ ≥ *c*_0_ · *r*_*a*,*b*_ these atoms are considered to be nonclashing (*f*(*a*, *b*) = 0). If, however, *d*_*a*,*b*_ ≤ *c*_1_ · *r*_*a*,*b*_, the atoms have a complete clash (*f*(*a*, *b*) = 1). For *c*_1_ · *r*_*a*,*b*_ < *d*_*a*,*b*_ < *c*_0_ · *r*_*a*,*b*_ we regard the clash as partial (0 < *f*(*a*, *b*) < 1). We consider a model to be clashing if the sum of its clashes is greater than 1. For this work, we used *c*_0_ = 0.85 and *c*_1_ = 0.5.

$$f\left(a,b\right)=\{\begin{array}{ll} 0, & \mathrm{if}\;d_{a,b}\geq c_{0}\cdot r_{a,b}\\ \frac{d_{a,b}-c_{0}}{c_{1}-c_{0}}, & \mathrm{if}\;c_{1}\cdot r_{a_{1},a_{2}}\leq d_{a_{1},a_{2}}\leq c_{0}\cdot r_{a_{1},a_{2}}\\ 1, & \mathrm{if}\;d_{a,b}\leq c_{1}\cdot r_{a,b} \end{array}$$ 1

When analyzing the rotamer states of multiple side chains, we distinguish between static and flexible atom clashes. Flexible atoms are all side chain atoms that might be moved due to a rotational operation, excluding backbone and the β-carbon atoms. When identifying rotamer clashes, we first check for clashes with static atoms, as these cannot be resolved through another rotation within our model. Any rotamer with such a clash is excluded from further consideration. In the second step, we evaluate the remaining rotamer combinations for clashes with other flexible atoms. If such a clash is detected, only the specific rotamer combination causing it is discarded.

#### Priority Scoring of Residues

Since the number of rotamer combinations grows exponentially with the number of rotated side chains, a meaningful selection of flexible side chains is crucial. To this end, we developed a novel efficient and heuristic method to identify rotamer multiplets that may undergo rotational changes upon binding. As input, we use pockets from a DoGSite3 ^46^ run, provided as both residue and atom PDB files. This information is subsequently used for excluding side chains that extend outward from the pocket.

Scoring of residues consists of two parts: First, residues are divided into static and flexible sets, with the latter being assigned a score representing their likelihood of rotating. Second, multiplets of flexible residues are constructed with respect to additional constraints on the number of rotational states and the number of residues.

Initially, only residues without potential rotamers are assigned to the rigid set, while the remaining residues are placed in the flexible set. Residues without potential rotamers are those that lack rotatable atoms in the pocket (rotatable atoms include side-chain atoms except for β-carbon and proline atoms). Then we calculate the solvent accessible surface area (SASA)^66^ for all rotatable side-chain atoms *SASA*_*sc*_. Subsequently, all possible rotamers for each side chain are generated and tested for clashes with static atoms. Rotamers with unacceptable clashes are discarded. For the remaining rotamers, pairwise RMSD values are calculated, and the maximum *RMSD*_*max*_ and mean *RMSD*_*mean*_ values are saved. A residue score *s*_*r*_ is then calculated as

$$s_{r}=SASA_{sc}\cdot \frac{n_{rotclashfree}}{n_{rot}}\cdot RMSD_{\max}\cdot RMSD_{mean}$$ 2

where *n*_*rot*_ is the number of initial rotamers and *n*_*rotclashfree*_ is the number of rotamers that have not been discarded. Since we are especially interested in rotations of hydrogen bond donors and acceptors, which might form crucial interactions with the ligand series, we define a minimum score for these residues *s*_*r*_*h*__ = min­(*s*_*hmin*_, *s*_*r*_) as long as they have more than one rotamer without unacceptable clashes.

Afterward, we use the user-supplied threshold *s*_*rigid*_ (default 1.0) and assign residues with *s*_*r*_ < *s*_*rigid*_ to the rigid set. Since this may increase the amount of clashes with static atoms, this procedure is repeated iteratively until no further side chains are added to the rigid set.

Side chains of the flexible set are then used for the identification of multiplets. Our goal is to identify groups of residues that potentially exhibit codependent movements enabling ligand binding. Side chains are considered potentially codependent if they have at least one rotamer combination that results in clashes or if the smallest atom distance between them is below a user defined threshold (default 1 Å).

Multiplets are sets of residues connected by potential codependency. Based on a user-specified maximum multiplet size, all resulting combinations or subsets are ranked by the sum of their residue scores. Highest scoring multiplets are written to an output file, which also includes a suggested number of rotamers to generate per side chain. This suggestion depends on a user-defined limit (default: 1000), the number of clash-free rotamer states, and the number of rotatable bonds specific to the amino acid type. By default, the number of rotamer states *n*_*rot*_ for a residue *r* of type *t*_*r*_ will be chosen according to eq 3. If the product of these values exceeds the overall limit, these values are reduced until the maximal number of combinations falls below the threshold.

$$n_{rot}\left(t_{r}\right)=\{\begin{array}{ll} 3, & \mathrm{if}\;t_{r}\in \left\{Cys,Ser,Thr,Val\right\}\\ 6, & \mathrm{if}\;t_{r}\in \left\{Asn,Asp,His,Ile,Leu,Phe,Trp,Tyr\right\}\\ 9, & \mathrm{if}\;t_{r}\in \left\{Arg,G\ln,Glu,Lys,Met\right\} \end{array}$$ 3

#### Generation of Rotamer Models

Rotamer models were generated by the SideChainRotator tool developed for this work. It creates rotational states based on an input protein structure provided as pdb or mmCIF file,^67,68^ a specified set of residues to rotate, and user-defined upper limits for the number of rotamer variants to generate.

Initially, all possible rotamers are applied independently to each specified residue and checked for clashes with static atoms. Rotamers with unacceptable clashes are discarded (see Protein Side Chain Clashes for details). Subsequently, a k-medioid clustering is performed on all remaining rotamers,^69^ using RMSD as the distance metric. The maximum number of rotamers to be generated (*k*) is determined by the user-specified input, which may stem from the values specified in the previous section.

After the selection of individual rotamer states, all possible combinations of rotamer states are constructed and checked for clashes between the flexible atoms. If a combination of rotamers exhibits unacceptable clashes, it is discarded; however, the individual rotamers are retained, as they may be compatible with other combinations.

Finally, all remaining rotamer states are sorted in descending order on the basis of their sum of acceptable clashes. Depending on the user-defined maximum number of output models, top-ranked models are written as pdb files.

Note that rotamer generation is possible, even if the rotated residue has missing atoms. In such cases, the missing atoms are repaired and the initial conformation is discarded entirely, leaving only the rotamers generated from the library.

### Assessment of Candidate Models

#### Docking

Our method is based on the small molecule docking of all ligands in a series into each structural candidate model. For this, we use the docking tool JAMDA,^41,45^ employing a softened clash term by reducing the zero point of the van der Waals (vdW) interaction from 0.95 to 0.9 times the sum of the vdW atomic radii. This adjustment accounts for uncertainties in the models and has proven beneficial for our approach. However, we expect that similar results are achievable using other docking tools like for example AutoDock Vina^70^ or Glide,^71,72^ since most state of the art docking tools tend to perform reasonably well in redocking tasks.^40,41^ Apart from the modified clash term and a specification for reduced output writing, we used JAMDA’s highly automated preparation pipeline and default parameters without further modifications. No target-specific parameter changes were performed. For all subsequent steps, only the top-scored pose for each model-ligand pair was used.

#### Calculating Consensus Scaffold Poses from a Docked Series

The top-scoring poses are processed by our newly developed pose clustering tool. The common molecular scaffold is supplied as a SMARTS^73^ expression, which is applied to each ligand pose to extract the scaffold poses. These scaffold poses are then clustered using the density-based clustering method DBSCAN.^54,74^

In this algorithm, data points are considered neighbors if their distance is smaller than a specified parameter ϵ. A data point is classified as “dense” if it has at least *n*_*c*_ neighbors. A cluster in the DBSCAN algorithm consists of connected dense points and all its neighbors. Every element not part of a cluster is labeled as noise, and it is not guaranteed that any cluster is detected. Regarding the task at hand—identifing protein structure models that allow for consistent scaffold placement—the algorithm generates clusters of scaffold poses, which are subsequently scored. Structures that do not yield any clusters are immediately discarded.

We use the RMSD of all scaffold atoms as the distance measure. Our default value for ϵ is 1 Å, which consistently delivered dense scaffold clusters with little variance. However, for the PDE10 case, we manually increased it to 2 Å due to the nearly symmetric scaffold, which resulted in 180 deg flips that inflated the RMSD. Enlarging ϵ in this way allows for flipped scaffolds with identical interaction patterns to be placed in the same cluster. For *n*_*c*_, we took 20% of the total number of scaffold poses.

For each cluster, we save a representative scaffold pose defined as the pose with the smallest maximum distance to all cluster members. This representative pose is considered the proposed positioning of this cluster and is used for subsequent performance analysis.

### Comparing Results with Experimental Structures

To compare the predicted models with experimental reference structures, we developed an additional tool for the binding site comparison. Predicted models are first aligned to the experimental binding site using functionalities developed by Bietz et al.^75^ The symmetry-corrected RMSD between the representative scaffold pose and the experimental ligand substructure was then calculated. Additionally, we computed the binding site RMSD for the entire pocket as well as for individual side chains.

### Tool and Data Availability

All mentioned tools are available as part of the NAOMI ChemBio Suite, which is freely available for academic use and can be licensed for commercial use (https://uhh.de/naomi). Data sets are available at 10.25592/uhhfdm.18828.

### Data Sets

#### Fatty Acid Binding Protein 4 (FABP4)

Model input was a set of 17 FABP4 inhibitors with a triazolopyrimidinone core^76,77^ and activities ranging from p*K*_i_ = 4.65–8.07 () as well as a FABP4 crystal structure (pdb code: 7FVU, nonprotein residues removed) without the Met40/Ser53 induced fit effect observed in FABP4-triazolopyrimidinone cocrystal structures. FABP4 inhibitory activities were measured using displacement of a fluorescently labeled fatty acid.^78^ Results were assessed against the FABP4 cocrystal structure with compound fabp4_16 (pdb code: 7FYJ).

#### Phosphodiesterase 10A (PDE10)

The inhibitor data set comprised 59 congeneric ligands, all sharing a triazolopyrazine core, with activities spanning a range of pIC_50_ values from 5.18 to 9.53 (). PDE10 IC50 values were measured using a scintillation proximity assay, as previously described.^48^ As protein input models, we used five homology models of PDE10, which were generated with the MOE^79^ standard homology model procedure and employing the PDE5 protein structures with pdb codes 1t9s, 1tbf, 1xoz, 2chm, 2h44 as templates, respectively (). Results were assessed against the PDE10 cocrystal structure with compound pde10_15 (pdb code: 5SEP).

#### Adenylate Cyclase 10 (ADCY10)

A set of 15 congeneric ADCY10 inhibitors with an aminopyrimidine scaffold were extracted from ChEMBL covering pIC50 values from 5.79 to 7.55. This was achieved with the recently published Activity Finder tool, which we used to search for activity data matching targets from the data set published by Škrinjar et al.^12^ The protein input consisted of a BOLTZ-1 cofolding prediction model for the ADCY10 pdb code = 7OVD kindly provided by Peter Škrinjar.^62^ Since the 25 cofolding prediction models were structurally very similar we just used the one with the highest ranking_score (seed-3995477496_sample-0.pdb) as input for our approach. Results were assessed against the ADCY10 cocrystal structure with compound adcy10_13 (pdb code: 7OVD).

## Supplementary Material





## Notes

SMILES and biochemical activities of all data sets (CSV)
Flexibility prediction tables for PDE10 and ADCY10, Flexible ADCY10 side chains as pocket graphics, Precision-recall plots for ranking, Flexible side chains in GOLD runs; file also includes a link to a Tar archive at 10.25592/uhhfdm.18828, which contains the following content: Structure files for initial protein models (PDB); Ligand series for each target (SDF); Measured affinities for ligands (CSV); File containing analysis results (CSV); successful prediction models (PDB); Configuration files for GOLD analysis (CONF); Resulting poses for GOLD analysis (SDF); SMILES for all series molecules (CSV) (PDF)

## Abbreviations

ADCY10: Adenylate cyclase 10
CPU: Central processing unit
DBSCAN: Density-based spatial clustering of applications with noise
FABP4: Fatty acid binding protein 4
FXR: Farnesoid X receptor
IC_50_: Half-maximal inhibitory concentration
IFD-MD: Induced fit docking-molecular dynamics
MD: Molecular dynamics
MOE: Molecular Operating Environment
PDB: Protein Data Bank
PDE10: Phosphodiesterase 10A
RMSD: Root-mean-square deviation
SAR: Structure–activity relationship
SASA: Solvent-accessible surface area
SGSR: Scaffold-Guided Structure Refinement
SMARTS: SMILES arbitrary target specification
SMILES: Simplified molecular input line entry specification
