# Supplementary material for: Predicting Ligand Binding Modes by Scaffold-Guided Structure Refinement
Source: J Med Chem. 2026 Jul 24;69(15):17961–73. doi: 10.1021/acs.jmedchem.6c00089 (PMC13492369; doi:10.1021/acs.jmedchem.6c00089)
Supplement: Supplementary file 1 [file jm6c00089_si_002.pdf]

Supporting Information: Predicting Ligand Binding Modes by Scaffold-Guided Structure Refinement

Jonathan Pletzer-Zelgert,† Matthias Rarey,*,† and Bernd Kuhn*,‡

†*University of Hamburg, ZBH - Center for Bioinformatics, Albert-Einstein-Ring 8-10, 22761 Hamburg, Germany*

‡*Roche Innovation Center Basel, F. Hoffmann-La Roche Ltd., Grenzacherstrasse 124, 4070 Basel, Switzerland*

E-mail: matthias.rarey@uni-hamburg.de; bernd.kuhn@roche.com

Phone: +49 (40) 428387351; +41 (79) 4735513

Contents

PDE10 **S-2**

Table S1: List of sampled PDE10 sidechain multiplets S-2

ADCY10 **S-3**

Table S2: List of sampled ADCY10 sidechain multiplets S-3

Figure S1: All potentially flexible ADCY10 pocket residues S-3

Precision Recall Plots **S-4**

Figure S2: Precision recall plot FABP4 . . . . S-4

Figure S3: Precision recall plot PDE10 . . . . S-4

Figure S4: Precision recall plot ADCY10 . . . . S-5

Table S3: Precision of the ranking S-5

Sidechains considered flexible in different GOLD runs **S-6**

Table S4: Sidechains considered flexible in different GOLD runs S-6

Description of SI Tar Archives **S-7**

PDE10

Table S1: List of sampled PDE10 sidechain multiplets

PDE10 residue ids	# of conformers	Template id
[Ile701,Met703,Met704,Phe719]	[4,7,7,5]	1t9s
[Asn562,Gln566,Ser577,Thr578]	[5,8,2,3]	1t9s
[Asn562,Gln566,Ser577,Glu582]	[5,8,2,9]	1t9s
[Leu665,Met703,Phe719]	[4,7,5]	1t9s
[Glu582,Thr623,Leu627,Asp664]	[9,3,6,6]	1t9s
[Asn562,Gln566,Thr580,Glu582]	[5,8,3,8]	1tbf
[Thr623,Leu627,Phe629]	[3,6,4]	1tbf
[Phe686,Met703,Met704,Phe719]	[5,8,5,5]	1tbf
[Leu665,Val668,Met703,Phe719]	[4,3,8,5]	1tbf
[Thr623,Leu625,Asp664]	[3,6,6]	1tbf
[Phe686,Ile701,Met703,Phe719]	[5,5,8,5]	1xoz
[Ile701,Met703,Met704]	[5,8,4]	1xoz
[Leu665,Val668,Met703,Phe719]	[6,3,8,5]	1xoz
[Leu627,Leu665,Val668,Phe719]	[6,6,3,5]	1xoz
[Thr623,Leu627,Leu665]	[3,6,6]	1xoz
[Leu665,Val668,Met703,Phe719]	[6,3,9,6]	2chm
[Phe686,Met703,Val712,Phe719]	[6,9,3,6]	2chm
[Glu582,Thr623,Leu627,Asp664]	[7,3,6,6]	2chm
[His515,Asn562,Met581,Glu582]	[4,5,7,7]	2chm
[Asn562,Met581,Glu582,Thr623]	[5,7,7,3]	2chm
[Glu582,His585,Phe586]	[9,5,5]	2chm
[Glu582,His584,Phe586]	[9,6,5]	2h44
[Asn562,Gln583,His585,D624]	[5,8,5,5]	2h44
[Asn562,Ser579,Gln583]	[5,3,8]	2h44
[Ser563,Gln583,Asp624,Leu627]	[3,8,5,5]	2h44

ADCY10

Table S2: List of sampled ADCY10 sidechain multiplets

ADCY10 residue ids	# of conformers
[Arg176,Lys340,Met419,Lys451]	[5,5,6,6]
[Arg176,Gln179,Asn180,Lys451]	[5,8,4,6]
[Asn180,Arg416,Met419,Lys451]	[4,6,6,6]
[Arg176,Leu177,Lys340,Met419]	[5,4,5,6]
[Arg176,Phe338,Lys340,Met419]	[5,4,5,6]
[Arg176,Lys334,Phe336,Phe338]	[5,8,6,4]
[Asp47,Asp99,Lys144,Arg176]	[5,5,8,5]
[Arg176,Leu177,Asp339]	[5,4,6]
[Thr52,Gln409]	[3,9]
[Asn412,Arg416]	[6,6]

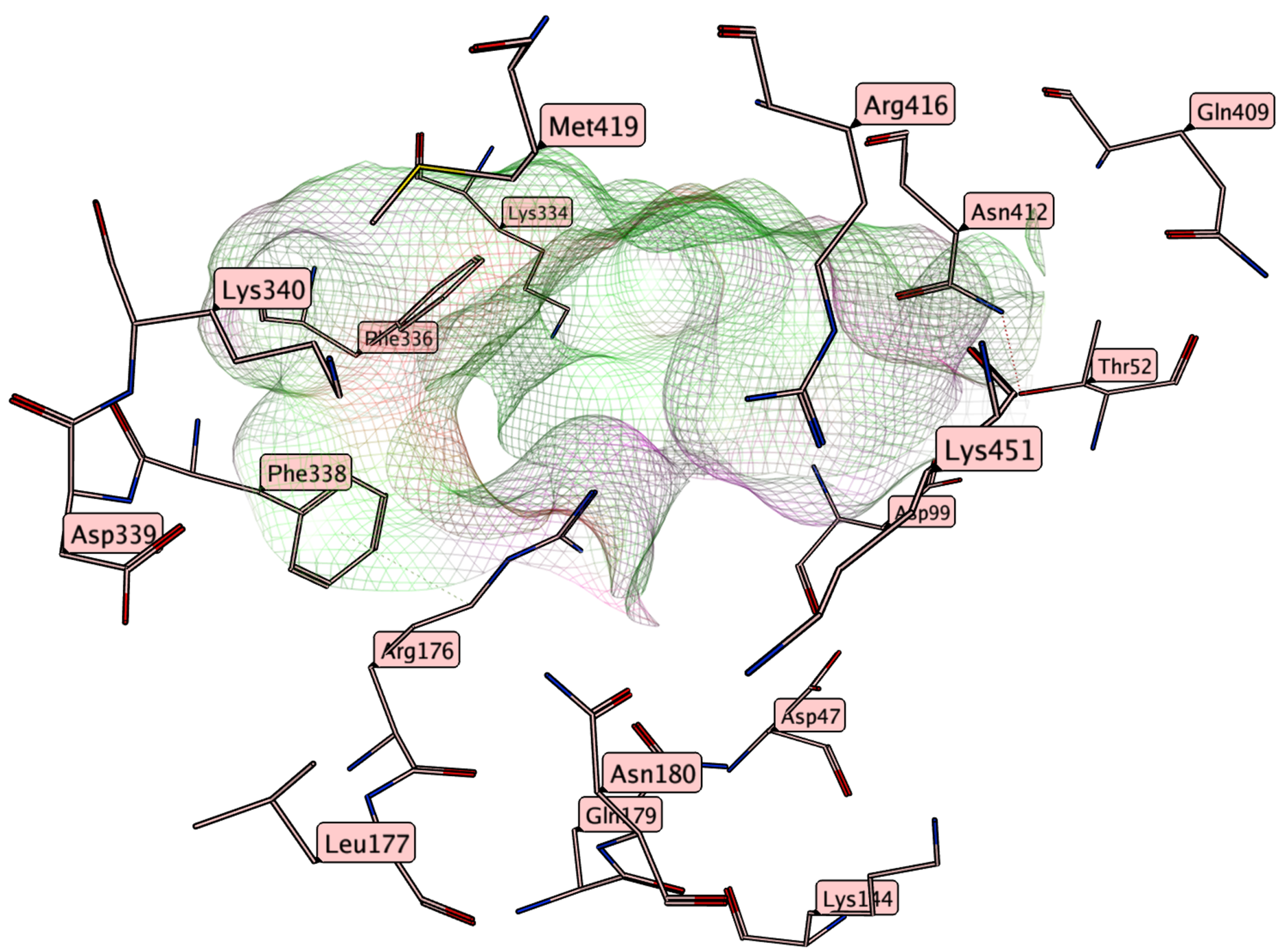

Figure S1: A depiction of all binding site residues of ADCY10, for which the flexibility analysis predicted a potential flexibility.

Precision Recall Plots

Table S3 lists success rates for the top 5, 20 and 50 models and the general percentage of models with correctly placed scaffold. Figures S2, S3 and S4 contain precision recall plots for each of the respective targets. While the recall describes the ratio of retrieved successful predictions when looking at an increasing amount of the ordered results, the precision is the amount of retrieved successful predictions divided by the total amount of retrieved predictions.

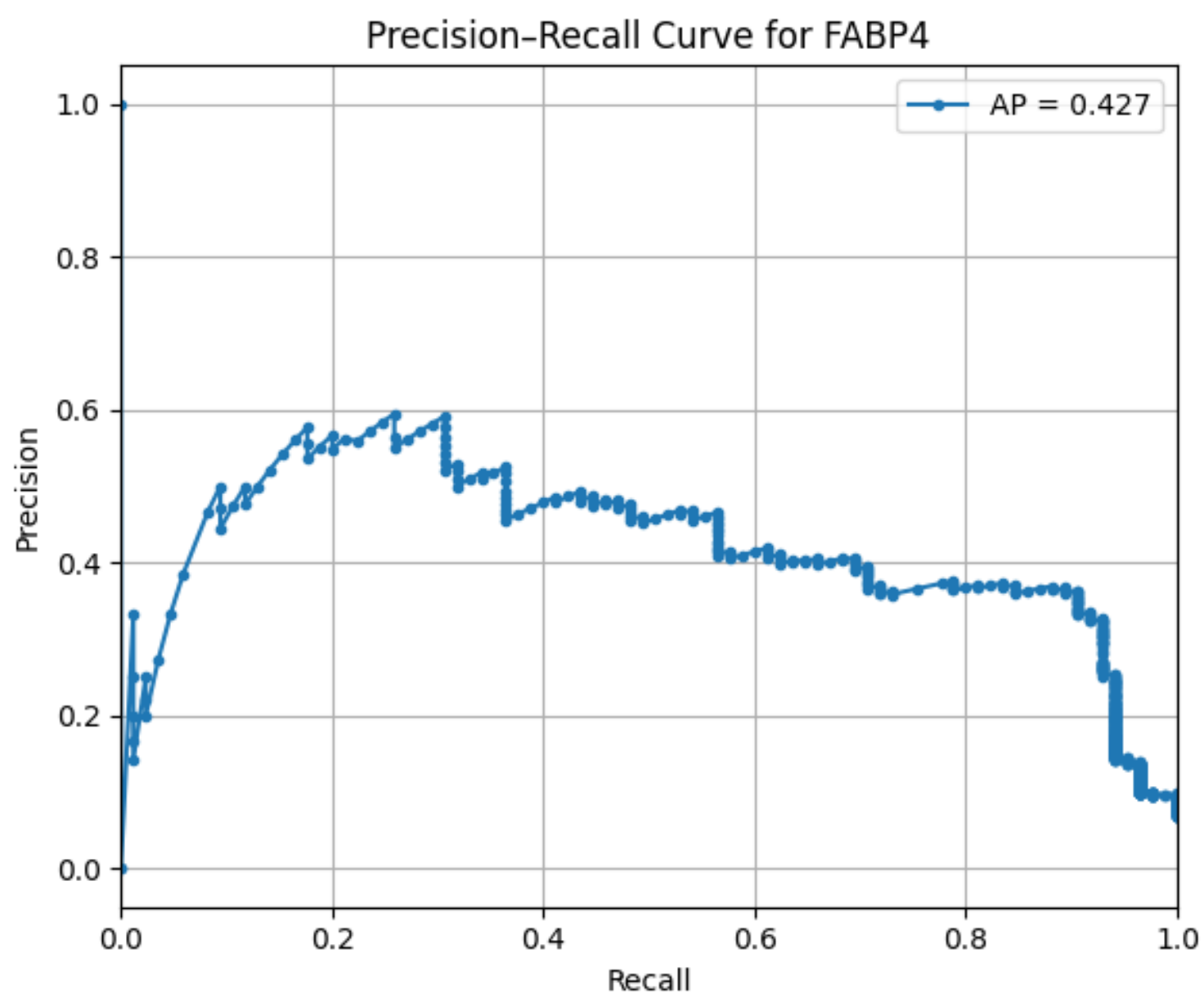

Figure S2: In case of FABP4 we achieve a reasonable enrichment of successful prediction. However top ranked models do include some negative cases with only one of the top 5 models being predicted correctly

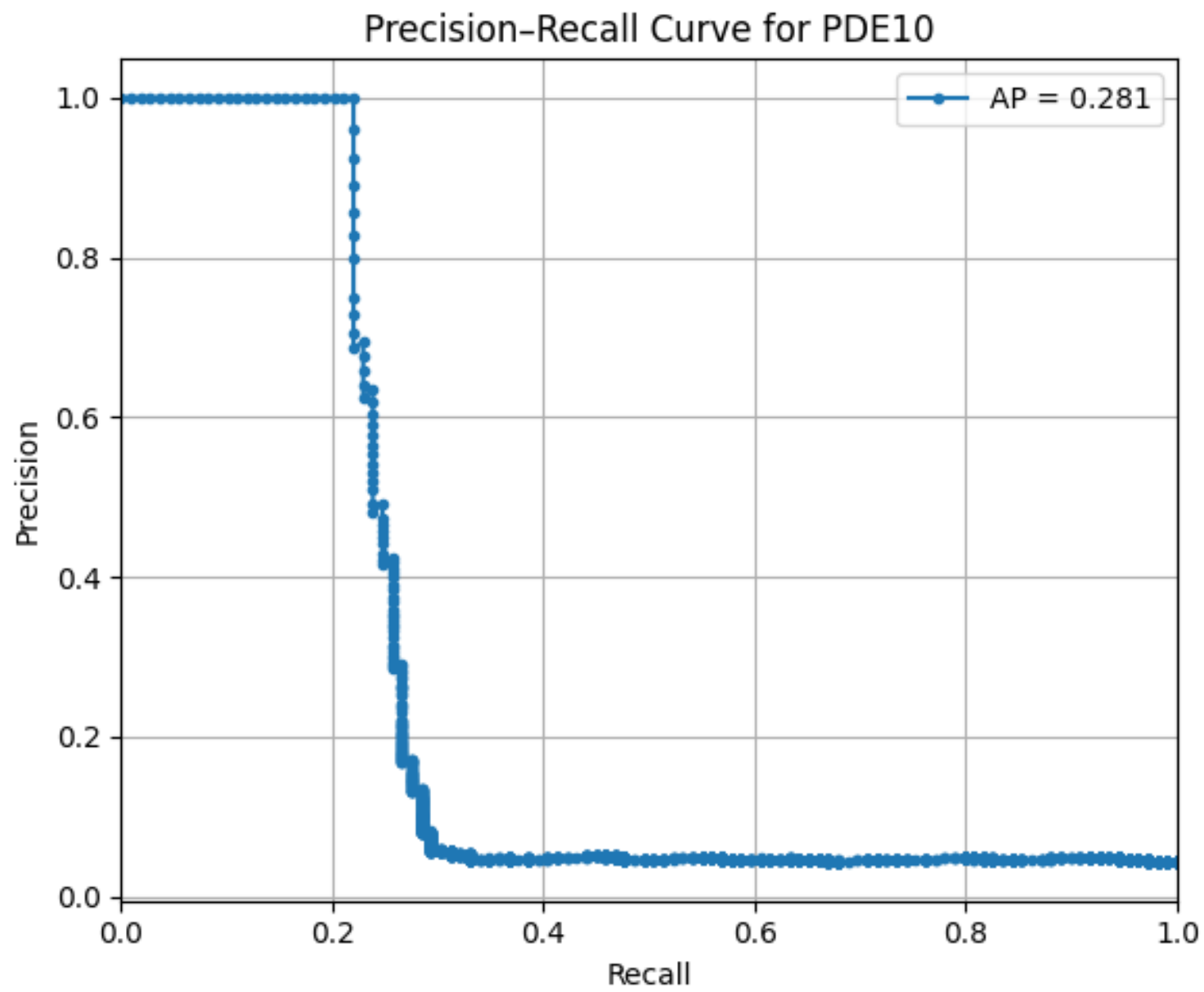

Figure S3: In the case of PDE10, we achieved a very successful separation of successful models. More than 20 positive models are ranked before all negative models

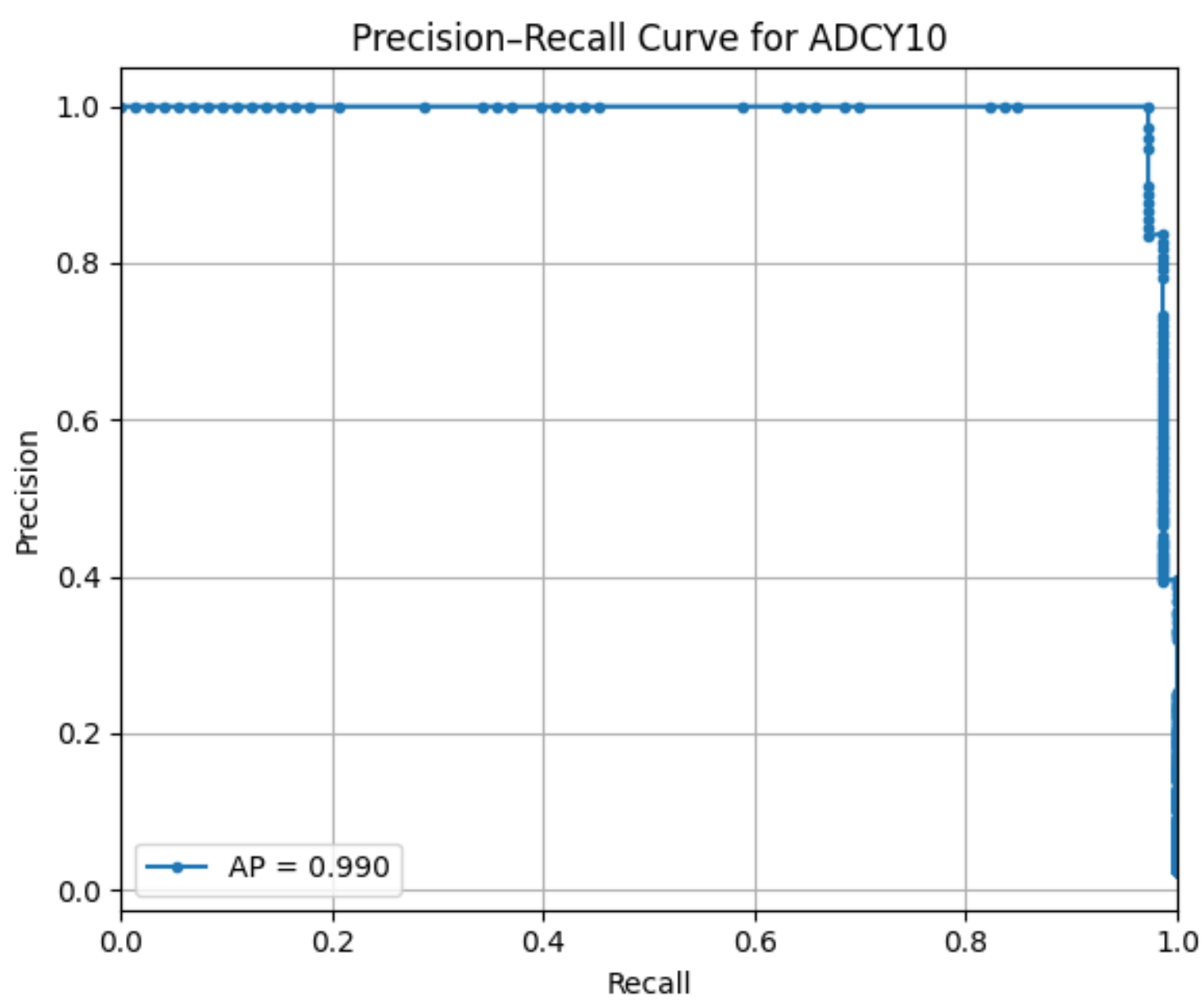

Figure S4: In the case of ADCY10, we achieved a near optimal separation of successful models. More than 90% of positive models have been ranked before all negative models

Table S3: Precision of the ranking for the k-top models

k	ADCY10	FABP4	PDE10
5	100%	20%	100%
20	100%	50%	100%
50	100%	52%	52%
Rate of good models	2.31%	6.55%	4.27%

The overall quality criterion used in this work was that a successful prediction can be found within the first 5 models since this is expected to be an amount that will in practice be visually inspected. This was achieved for all three targets.

Sidechains considered flexible in different GOLD runs

Table S4: Side chains considered flexible in different GOLD runs

	ADCY10 Broad	ADCY10 Focused	FABP4 Broad	FABP4 Focused	PDE10 Broad	PDE10 Focused
Side Chains Considered Flexible	Phe 45, Leu 94, Lys 95, Phe 96, Leu 101, Leu 102, Phe 165, Leu 166, Val 167, Ile 168	Arg 176, Lys 334, Phe 336, Phe 338	Met 40, Ile 51, Ser 53, Thr 60, Glu 72, Arg 78, Gln 95, Ile 104, Arg 106, Arg 126	Met 40, Ser 53, Thr 60	His 515, Leu 625, Asp 664, Leu 665, Val 668, Phe 686, Met 703, Met 704, Val 712, Phe 719	Met 703, Met 704, Phe 719

Description of SI Tar Archives

This data set can be found under `https://doi.org/10.25592/uhhfdm.18828`. It contains one archive directory for each of the three targets: FABP4, ADCY10 and PDE10. These directories will contain the following files:

- Initial models used as input (One for FABP4 and ADCY10, Five for PDE10).
- One file containing all series ligands in the SDF format.
- One csv file containing ligand names, ligand SMILES and measured activities.
- One csv file containing all information from the evaluation.
- One subdirectory containing the successfully identified model as a pdb file containing the protein, an SD file containing the docked ligand poses, as well as one Mol2 file containing the representative scaffold for the cluster.
